# Thymic output in human newborns is shaped by environmental exposures and a common TCRD genetic variant

**DOI:** 10.70962/jhi.20260003

**Published:** 2026-05-04

**Authors:** Ziyang Tan, Camille Kergaravat, Laura Gonzalez, Anette Johnsson, Erika Negrini, Christian Pou, Anna Karin Bernhardsson, Hugo Barcenilla, Margarita Ivanchenko, Yang Chen, Ewa Henckel, Tadepally Lakshmikanth, Jaromír Mikeš, Anna James, Agata Cieslak, Vahid Asnafi, Jonathan Desponds, Magnus Fontes, Magali Irla, the Milieu Interieur Consortium, Laurent Abel, Andres Alcover, Hugues Aschard, Philippe Bousso, Nollaig Bourke, Petter Brodin, Pierre Bruhns, Nadine Cerf-Bensussan, Ana Cumano, Christophe D'Enfert, Ludovic Deriano, Marie-Agnès Dillies, James Di Santo, Gérard Eberl, Jost Enninga, Jacques Fellay, Ivo Gomperts-Boneca, Milena Hasan, Gunilla Karlsson Hedestam, Serge Hercberg, Molly A. Ingersoll, Olivier Lantz, Rose Anne Kenny, Mickaël Ménager, Frédérique Michel, Hugo Mouquet, Cliona O'Farrelly, Etienne Patin, Antonio Rausell, Frédéric Rieux-Laucat, Lars Rogge, Magnus Fontes, Anavaj Sakuntabhai, Olivier Schwartz, Benno Schwikowski, Spencer Shorte, Frédéric Tangy, Antoine Toubert, Mathilde Touvier, Marie-Noëlle Ungeheuer, Christophe Zimmer, Matthew L. Albert, Darragh Duffy, Lluis Quintana-Murci, Darragh Duffy, Etienne Patin, Lluis Quintana-Murci, Emmanuel Clave, Antoine Toubert, Petter Brodin

**Affiliations:** 1 Unit for Clinical Pediatrics, Department of Women’s and Children’s Health, Karolinska Institutet, Solna, Sweden; 2 Institut de Recherche Saint Louis, INSERM UMRS 1342, Université Paris Cité, Paris, France; 3 Laboratoire d’Onco-Hematologie, Hôpital Necker Enfants Malades, AP-HP, Paris, France; 4 CNRS, INSERM U1151, Institut Necker Enfants Malades (INEM), Université Paris Cité, Paris, France; 5 Institut Roche, Boulogne-Billancourt, France; 6 CNRS, INSERM, CIML, Centre d'Immunologie de Marseille-Luminy, Aix Marseille University, Marseille, France; 7 Translational Immunology Unit, Institut Pasteur, INSERM U1223, Université Paris Cité, Paris, France; 8 Human Evolutionary Genetics Unit, Institut Pasteur, CNRS UMR2000, Université Paris Cité, Paris, France; 9 Chair Human Genomics and Evolution, Collège de France, Paris, France; 10 Laboratoire d’Immunologie et d’Histocompatibilité, Hôpital Saint‐Louis, AP‐HP, Paris, France; 11 Medical Research Council Laboratory of Medical Sciences (MRC LMS), Imperial College Hammersmith Campus, London, UK; 12 Department of Immunology and Inflammation, Imperial College London, London, UK

## Abstract

Naïve T cell output from the thymus varies across the human lifespan and is a key determinant of health, differing between individuals by age, sex, and genetics. How thymic output is dynamically regulated early in life in response to initial microbial colonization remains unclear. We report longitudinal thymic output dynamics, measured as T cell receptor excision circles (TRECs), in 136 newborns from Stockholm, Sweden. Thymic output increases after birth following initial microbial colonization, peaking at 3–4 mo. Peak height correlates with plasma levels of RANKL and lymphotoxin-α and with a common genetic variant in the TCRD locus previously linked to adult thymopoiesis. B cell lymphopoiesis measured by KRECs reveals divergent dynamics between B and T cell branches of the adaptive immune system in early life. Findings are corroborated by thymic tissue analyses, in which local RANKL secretion correlates with medullary, but not cortical, epithelial cell numbers. These results illuminate the establishment of healthy immune–microbe interactions in early human life.

## Teaser

Longitudinal profiling of 136 newborns shows thymic output peaks at 3–4 mo, correlating with plasma RANKL, lymphotoxin-α, and the TCRD variant rs2204985. In thymic tissue, RANKL tracks medullary—but not cortical—epithelial cellularity, suggesting a microenvironmental basis for thymopoiesis.

## Introduction

The thymus reaches its peak size and function during late fetal development and early infancy, serving as the primary site for T cell development and maturation. During this critical period, the thymus produces a diverse repertoire of naïve T cells through a complex process of positive and negative selection that establishes the foundation of adaptive immunity, as well as immune tolerance through the generation of regulatory T cells (Tregs). In healthy newborns, thymic output is remarkably high, with estimates suggesting the production of >10^9^ new T cells daily (1), and these naïve T cells are functionally heterogeneous (2, 3). In children thymectomized early in life, numbers of naïve T cells are greatly reduced and their function less diverse at 5 years of age, but thymic tissue regeneration later in life can partially restore the diversity of the naïve T cell compartment (2). Maternal health status and stress hormones during delivery have been reported to hamper thymic output in newborn mice (4) and human newborns (5). Changes in thymic output in response to postnatal environmental exposures are poorly understood.

**Figure S1.**
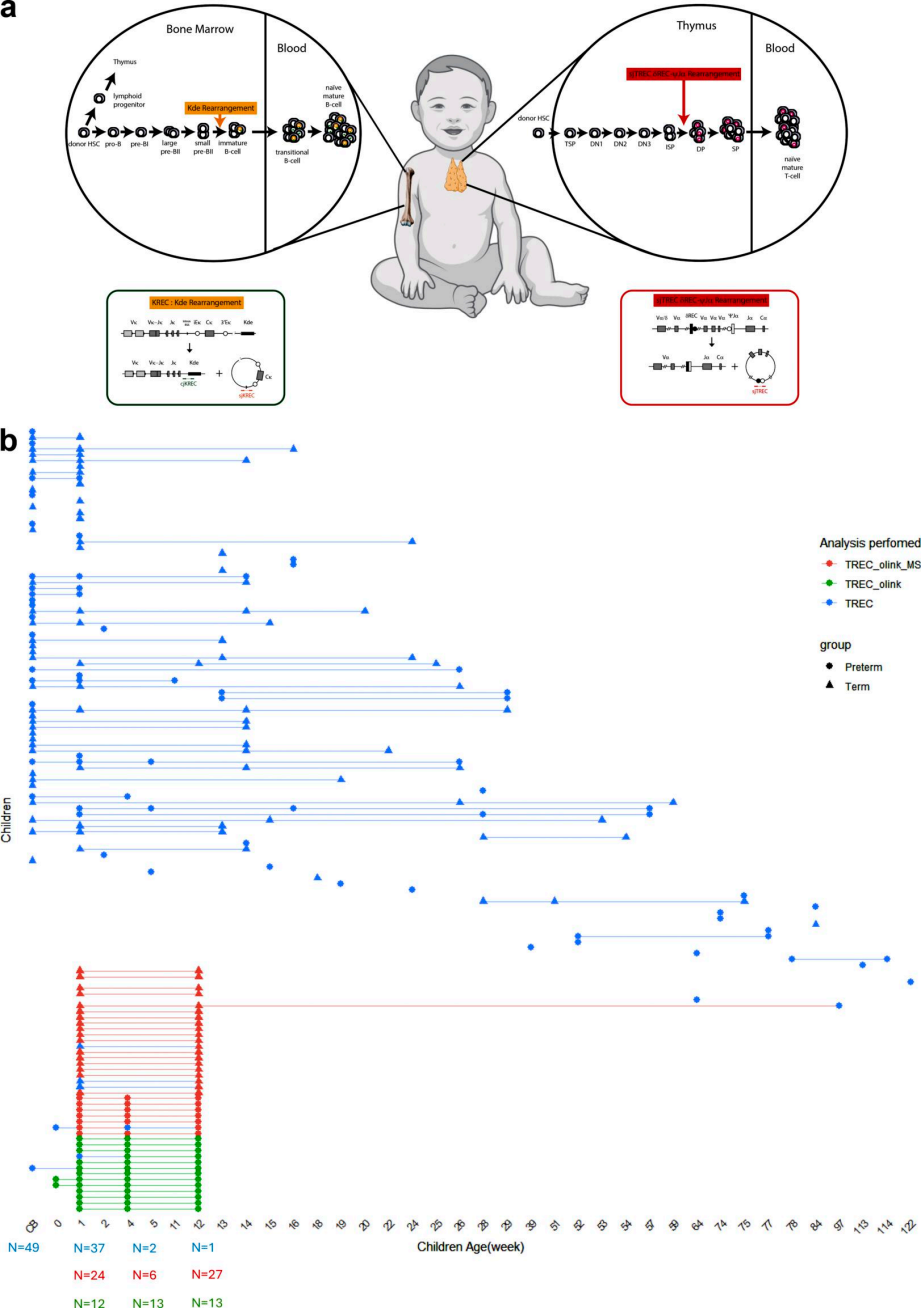
**Cohort design and TREC analysis description. (a)** Scheme of TREC and KREC assay. Right: sjTREC are generated in the thymus during T cell differentiation, between the ISP and DP stages. It corresponds to the excision of the delta locus excision between the δREC and the ΨJα segments. This rearrangement occurs in around 80% of αβ T cells. sjTRECs are small episomal circular DNA without an origin of replication, so they are not replicated during subsequent T cell division and are directly proportional to thymic T cell production. Left: KREC are generated in the bone marrow during B cell differentiation, between Small preB and immature B stages. It corresponds to the invalidation of the nonfunctional κ chain, by excision of the Cκ segment between the intron recombination signal sequences (RSS) and the κde elements. This rearrangement occurs in around 30% of κ^+^ cells and in 100% of λ^+^ cells (around 50% of all B cells). Like sjTREC, sjKREC are not replicated and are directly proportional to B cell production. CjKREC are on the genomic DNA and are so proportional to the total number of B cells; log2(CjKREC/sjKREC) corresponds to the mean number of B cell division since the K invalidation. HSC, hematopoietic stem cell; TSP, thymic seeding progenitor; DN, double negative; ISP, immature single positive; DP, double positive; SP, single positive. **(b)** Summary of samples and type of analysis included in the study according to the children’s age. *N* represents number of samples according to analysis performed and the shape of the point to the preterm or term group.

Thymic output gradually declines with age, beginning in early childhood and continuing throughout life, although the thymus maintains some functional capacity even in adulthood. A powerful method to track thymic output is the analysis of T cell receptor excision circles (TRECs), first discovered in the late 1980s as circular DNA fragments generated during T cell receptor gene rearrangement in developing T cells (6, 7) (Fig. S1 a). Their significance for clinical applications was recognized in the late 1990s when researchers found that TRECs could serve as reliable markers of recent thymic emigrants (8). A pivotal development occurred in 2005 when researchers demonstrated that TREC analysis could be performed using dried blood spots from newborns to detect severe combined immunodeficiency (SCID) (9). In 2008, Wisconsin became the first US state to implement TREC-based newborn screening for SCID, following validation studies showing that this approach could effectively identify infants with various forms of primary immunodeficiency. TREC screening is now a standard component of newborn screening programs in the United States and many other countries and has led to improved detection and treatment outcomes for infants with SCID and other T cell lymphopenic immunodeficiencies (10).

By carrying out signal-joint TREC (sjTREC) quantification in the peripheral blood of healthy adults, we previously identified, in addition to strong age and sex-dependent differences, a genetic control of human thymopoiesis at the *TCRA-TCRD* locus between *DD2–DD3* gene segments (genetic variant *rs2204985*) (11). However, it remains unknown whether this genetic variation impacts thymic function in newborns. Similar to TREC generation, DNA excision circles generated during immunoglobulin K-chain rearrangements (κ-deleting recombination excision circles, or KRECs) have been developed to evaluate B cell generation (12) (Fig. S1 a), the monitoring of which has been proposed alongside that of TRECs in neonatal screening for SCID (13).

Here, we analyze thymic output and B cell neogenesis in a cohort of healthy children born in Stockholm, Sweden, sampled frequently during their first months and years of life (14, 15). We find an initial surge in thymic output, significantly impacted by a recently described common genetic variant *rs2204985*, as well as gestational age at birth, but not impacted by mode of delivery or sex. B cell neogenesis, however, was found to be independent of these parameters. We also describe correlates of thymic output in the developing postnatal immune system of human newborns.

## Results

### Cohort description and multiomic profiling of immune development

**Figure 1.**
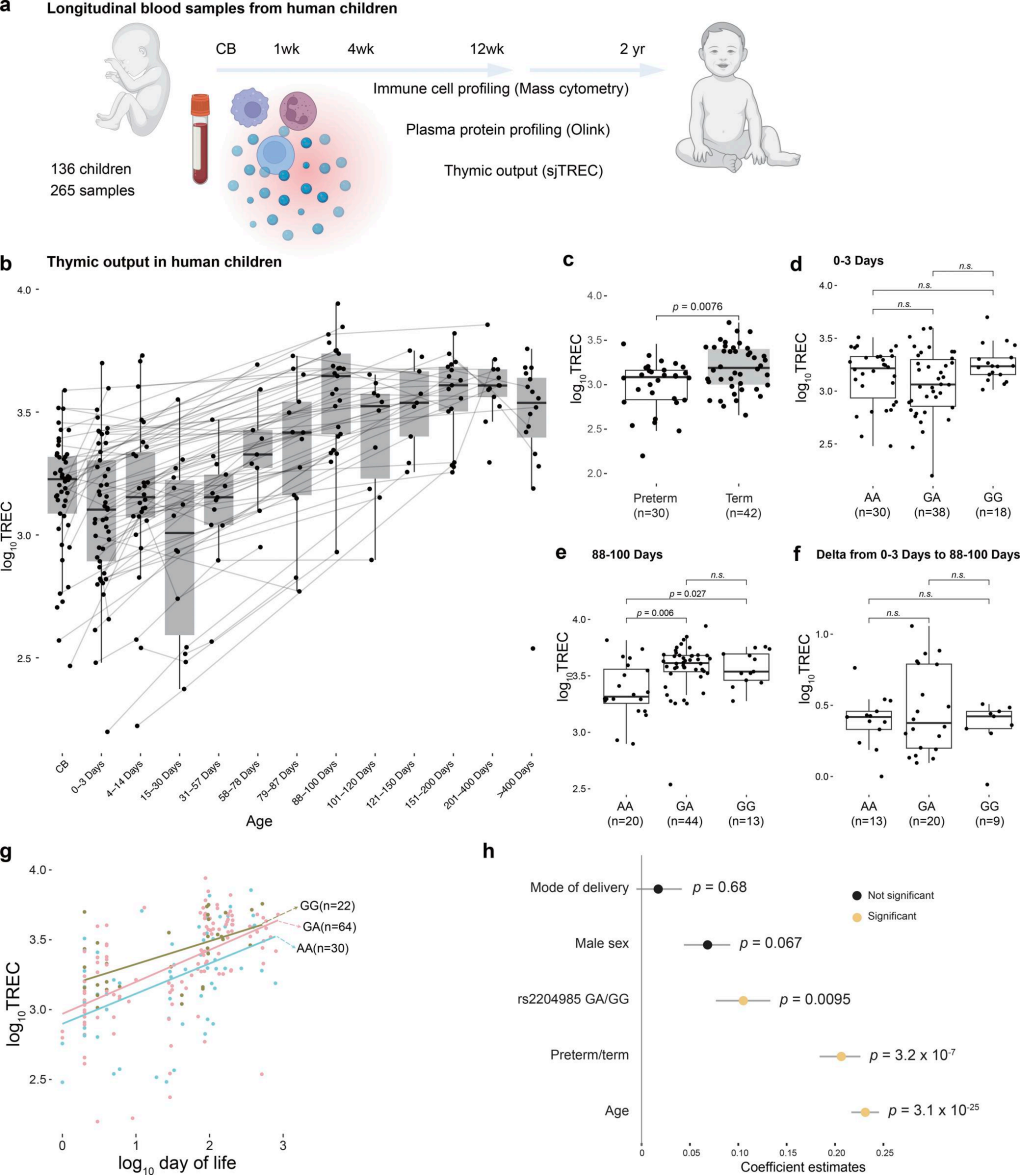
**Thymic output in newborn children. (a)** Cohort overview and samples collected and analyses performed. **(b)** Longitudinal changes of DNA samples showing a surge of thymic output 3 mo after birth in healthy human newborns. **(c)** Thymic output levels of preterm and born at term children in the first week of life. P values are from Wilcoxon test. **(d)** Thymic output of children at 0–3 days of life. **(e)** Thymic output of children at 88–100 days of life. **(f)** The delta values of thymic output for each child between 88–100 days and 0–3 days of life. P values are from Wilcoxon test and adjusted with Benjamini–Hochberg method. P values >0.05 are shown as n.s. **(g)** Postnatal monitoring of thymic output levels separated by rs2204985. All samples across time points of each genotype are used in the regression line. **(h)** Coefficient estimates from generalized linear model predicting thymic output using the listed variables. Grey lines represent 95% confidence intervals from 500 bootstrap iterations. P values are from ANOVA test of the model.

The cohort contains 265 longitudinal whole blood samples from 136 children born preterm (*n* = 68) or at term (*n* = 68) at Karolinska University Hospital between 2016 and 2019 (Fig. 1 a). Whole blood samples were collected at birth (cord blood), week 1, week 4, week 12, and longitudinally up to 2 years of age. We performed mass cytometry using a panel of 40 antibodies targeting markers of activation and differentiation and characterized 14 immune subpopulations. Plasma protein profiles were described using Olink assays (16) (Olink) detecting a panel of 76 proteins. sjTRECs and KRECs were measured to quantify thymic output and B cell generation respectively. The detailed schedule of sampling and analysis in the cohort is presented (Fig. S1 b).

### sjTREC analysis of thymic output

**Figure S2.**
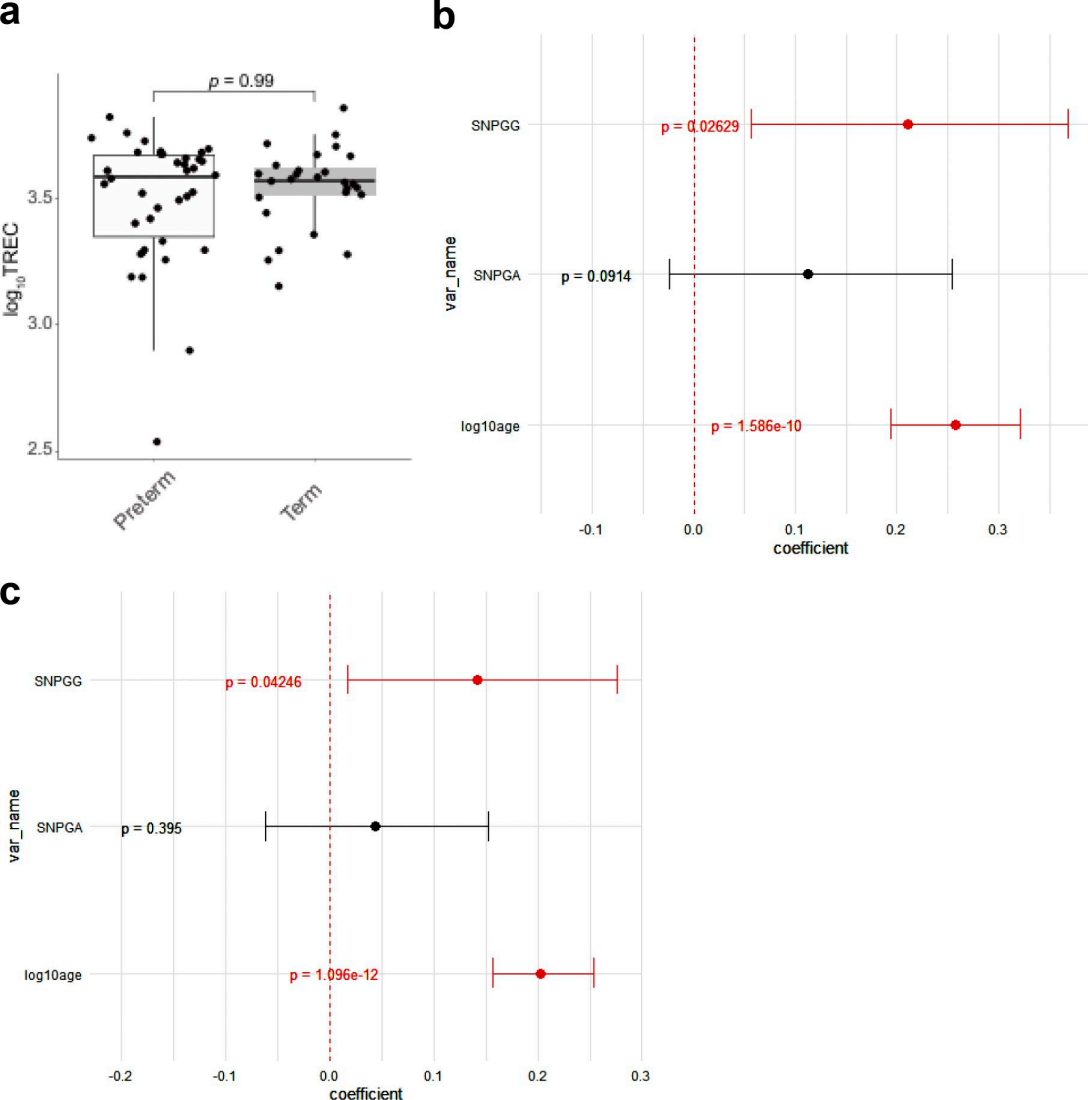
**Thymic output in preterm and term delivered children. (a–c)** Thymic output in preterm (*n* = 34) and in term (*n* = 23) children after 100 days of life. Coefficient estimates from generalized linear model predicting thymic production using the listed variables in preterm (b) and term (c) born children. Grey lines represent 95% confidence intervals. P values are from the generalized linear model.

The thymic output of newborn children, unlike that of healthy adults, increased with every day of life for the first 100 days and reached a plateau thereafter (Fig. 1 b). A surge in thymic output was observed around 3 mo after birth. Confirming previous observations (17, 18, 19), the difference between children born at term and preterm was significant within the first week (Fig. 1 c). However, this difference disappeared after 100 days (Fig. S2 a), following the pattern of other postnatal immune changes as previously described (14, 15). The genotypes of the *rs2204985* variant, which have been reported to influence thymic output in healthy adults (11), also affect the thymic output of newborn children following the same trend as observed in adults. No difference in thymic output was observed at birth (0–3 days) between AA, GA, and GG genotypes (Fig. 1 d). However, children with GA and GG genotypes had significantly higher thymic output compared with AA later in life (88–100 days, Fig. 1 e). No difference was observed in the delta value between 0–3 days and 88–100 days, probably due to limited statistical power (Fig. 1 f). While investigating the longitudinal changes, children with the GG genotype have the highest thymic output, followed by GA and then AA (Fig. 1 g). The contributions made by mode of delivery, sex, *rs2204985*, gestational age at birth, and age (log_10_ transformed) were modelled using a generalized linear model (Fig. 1 h). According to the model, postnatal thymic output is significantly impacted by the *rs2204985* variant (P = 0.0095), preterm or term birth (P = 3.2 × 10^−7^), and postnatal age (P = 3.1 × 10^−25^), but not mode of delivery or sex. In the model, a higher thymic output is associated with the GG genotype, mature birth and older age. Additionally, we have analyzed the association between sjTREC levels and *rs2204985* genotype in born at term and preterm children separately, considering age in a generalized linear model (Fig. S2, b and c). We confirm the association between *rs2204985* genotype and TREC levels in the cohort both for preterm (P = 0.02) and born at term children (P = 0.042).

### Peripheral blood cell and protein correlates of thymic output

**Figure 2.**
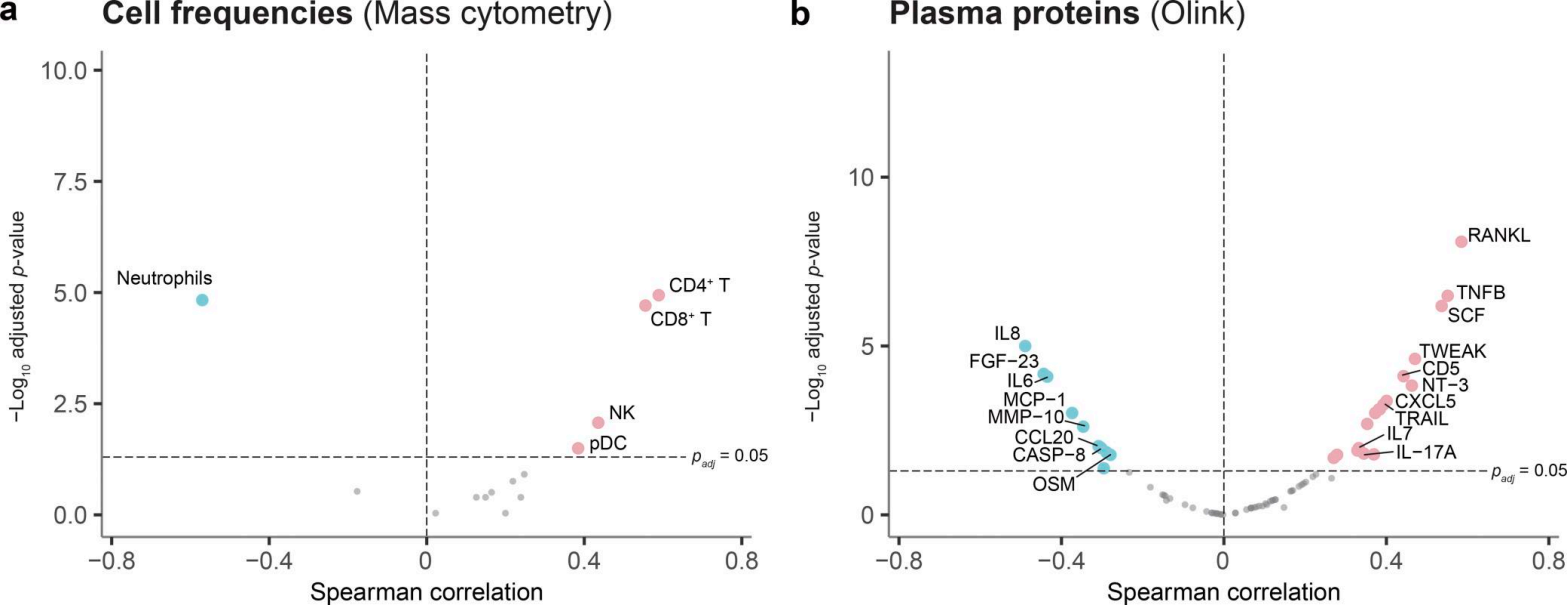
**Associations between thymic output and blood immune profiles. (a)** Spearman correlation between thymic output (TREC) and frequencies of 14 immune subpopulations characterized by mass cytometry. **(b)** Spearman correlation between thymic output levels and relative protein concentration levels (NPX) of 76 proteins characterized by Olink Target 96 inflammation panel. P values are corrected using Benjamini–Hochberg adjustment.

**Figure S3.**
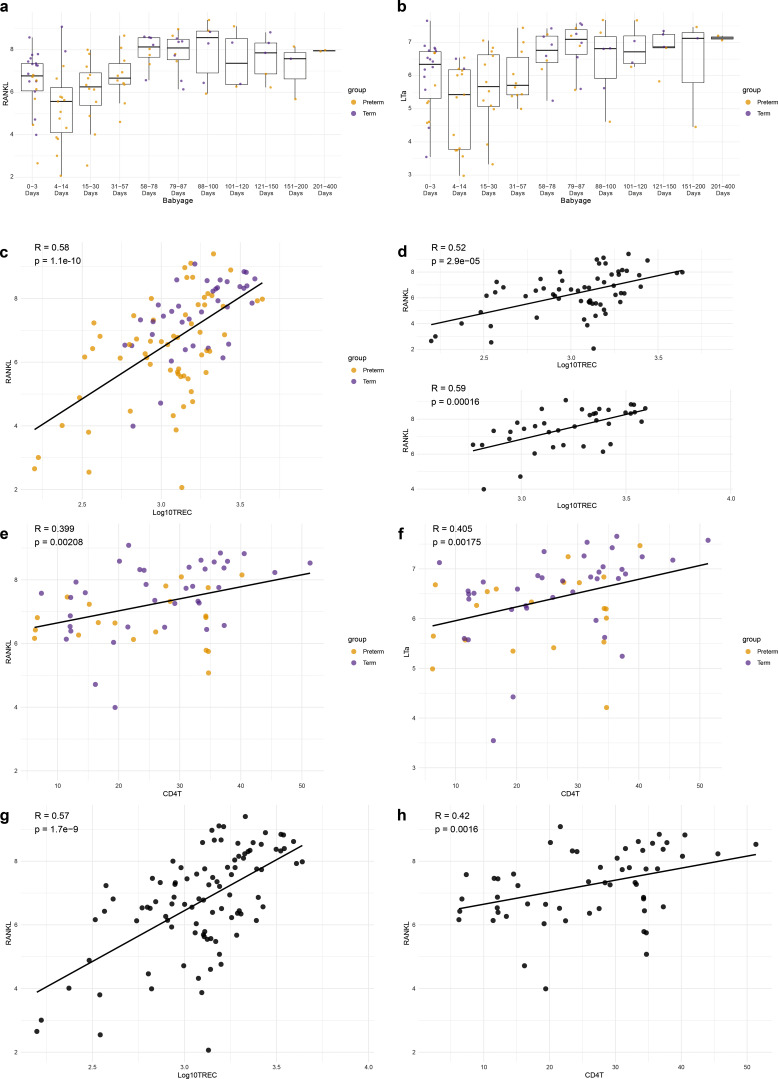
**RANKL and LTα correlation with thymic output and cell population independently of delivery status and steroids administration. (a–h)** Relative protein concentration (NPX) of RANKL (a) and LTα (b) according to delivery status and age group. Spearman correlation of RANKL and thymic output in all cohort (c) or separated by term status (d). Spearman correlation of CD4 T cells percentages with RANKL (e) and LTα (f) colored by delivery status. Correlation of RANKL levels and sjTREC (g) or CD4 frequency (h) in children who did not received steroids.

Due to the dynamic and heterogenous thymic output levels of newborn children, we investigated their associations with other immune characteristics. The frequencies of CD4^+^ T cells, CD8^+^ T cells, natural killer (NK) cells, and plasmacytoid dendritic cells (pDCs) show a positive correlation with thymic output, while neutrophils show a negative correlation (Fig. 2 a). Associations with CD4^+^ T cells, CD8^+^ T cells, and neutrophils are consistent with results observed in adults, but the NK and pDC associations suggest a potential age-specific regulation. Among the plasma proteins, RANK ligand (RANKL), and TNFB (or lymphotoxinα, LTα) were most strongly correlated with sjTREC levels (Fig. 2 b). Individual data points according to age for RANKL and LTα are shown in Fig. S3, a and b. RANKL and LTα are both potent inducers of medullary thymic epithelial cell (TEC) (mTEC) growth and AIRE^+^ mTEC differentiation (11, 20). In mice, the administration of RANKL can boost thymic regeneration, both after bone marrow transplantation (21) as well as in aged mice (22). RANKL also increases TEC cellularity in human organotypic cultures (22). Here, we show that the levels of circulating RANKL and LTα correlate strongly with thymic output in healthy newborns. The association between RANKL and sjTREC levels is significant in both preterm children (P = 2.9.10^−5^) and in children born at term (P = 0.00016) (Fig. S3, c and d). There is no significant difference in serum RANKL and LTα levels according to rs2204985 genotype. Conversely, we observed a negative correlation between sjTREC levels and inflammatory mediators such as IL-8, oncostatin M, and IL-6. IL-8 expression was previously associated with preterm birth in children (15). In normal human thymi, elevated levels of IL-6 and oncostatin M have been associated with decreased thymic function and sjTREC levels (23). RANKL and LTα levels also correlate with CD4 T cells frequencies (Fig. S3, e and f), CD4 T cells being one of the main RANKL producers. As steroid administration could potentially impact RANKL levels, we validated the correlations between RANKL, sjTRECs levels, and CD4 frequencies in the group of children who did not receive this treatment (Fig. S3, g and h). Taken together, these findings could reflect the influence of the environment on thymic function in newborns.

### B cell development in newborns

**Figure 3.**
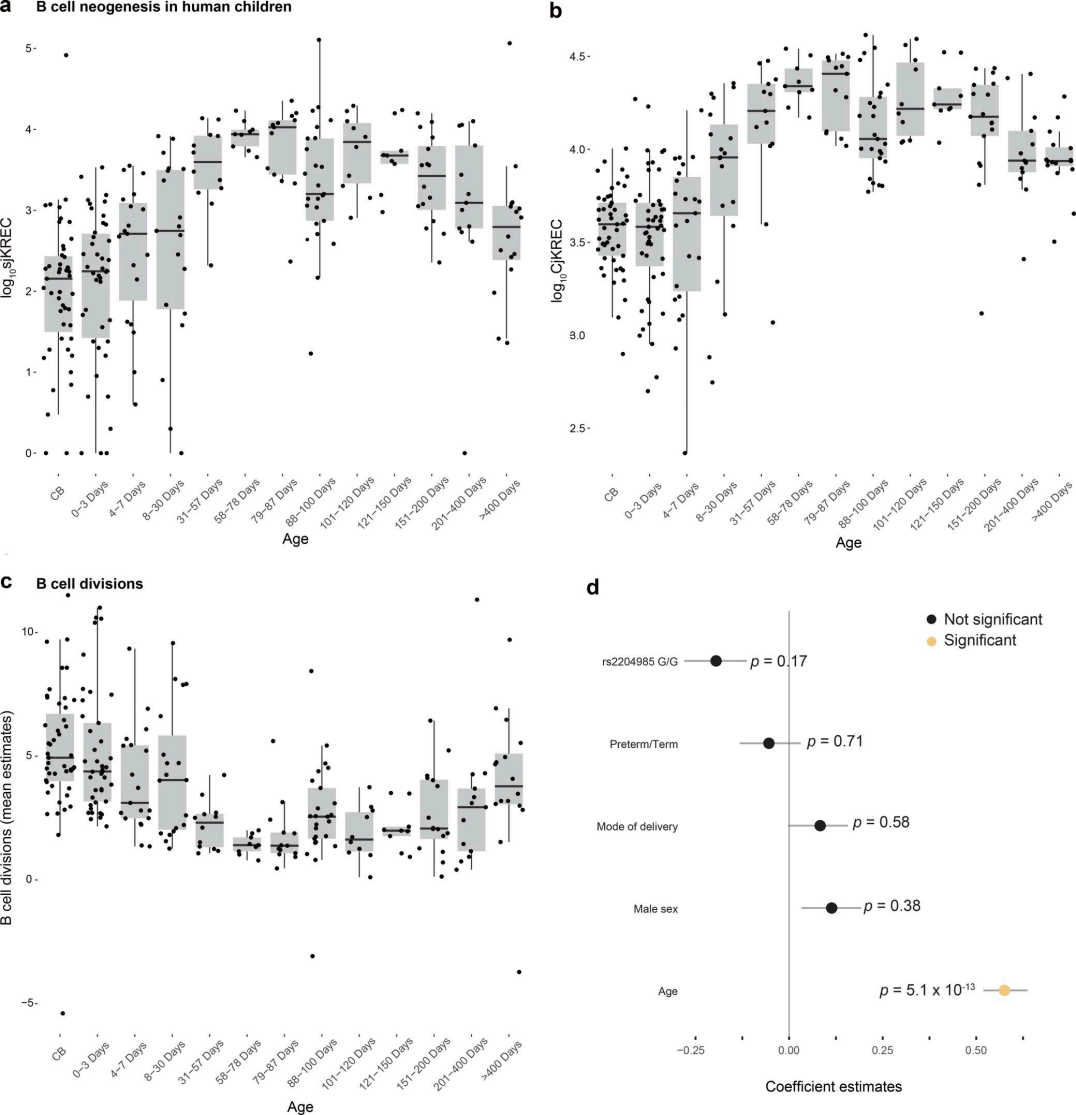
**B cell neogenesis in newborn children. (a)** Longitudinal monitoring of sjKREC levels in the blood of newborn children showing a surge 2 mo after birth, earlier than their thymic outputs. **(b)** CjKREC levels in the blood of newborn children. **(c)** Mean number of B cell divisions estimated by log2(CjKREC/sjKREC). **(d)** Coefficient estimates from generalized linear model predicting B cell generation using the listed variables. Grey lines represent 95% confidence intervals from 500 bootstrap iterations. P values are from ANOVA test of the model.

B cell generation can be studied by quantification of KRECs generated in the bone marrow during B cell development (12). The coding joint (Cj) of this rearrangement is duplicated during each cell division, whereas the signal joint KREC (sjKREC) remains stable as episomal DNA (Fig. S1 a). Therefore, in the periphery, CjKRECs and sjKRECs reflect total and recently produced B cells, respectively, whereas the ratio between Cj and sjKRECs is an indication of the mean number of divisions achieved by B cells following K-chain invalidation. sjKREC levels followed the same general pattern as sjTRECs; however, they reached a plateau during the second month after birth (Fig. 3 a), which is earlier than that of sjTRECs. This could be explained by differences in T and B cell development, with T cells requiring an additional and specific step in the thymus to differentiate from circulating bone marrow-derived progenitors. Newly generated B cells underwent a high division rate during the first month after birth to raise total B cell levels as reflected by CjKRECs (Fig. 3, b and c). Neither gestational age at birth nor the *rs2204985* SNP had any influence on B cell development (Fig. 3 d).

### Postnatal thymic tissue analysis

**Figure 4.**
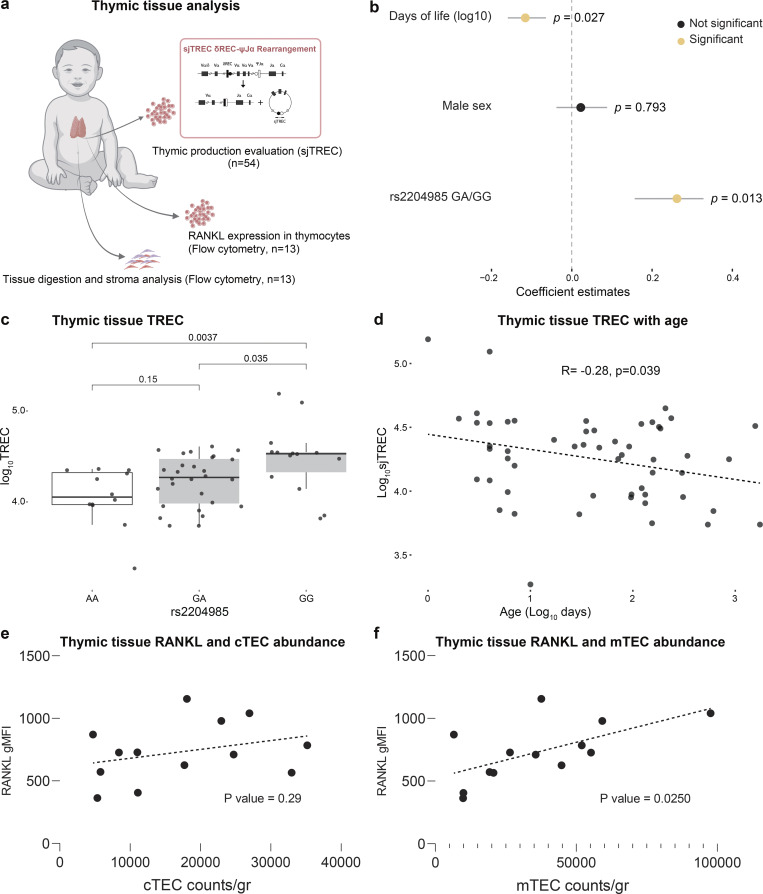
**Thymopoiesis measured in thymus tissue from young children. (a)** Thymus samples collected from children undergoing surgery and subject to TREC analysis. **(b)** Coefficient estimates from a generalized linear model predicting thymopoiesis (TREC) levels taking variables age, sex, and rs2204985 status into account. Grey lines represent 95% confidence intervals for coefficients. P values are from ANOVA tests. **(c)** Thymopoiesis (TREC) levels in newborn children separated by their genotypes for SNP rs2204985 (AA: *n* = 11, GA: *n* = 29, GG: *n* = 14). P values are from Wilcoxon pairwise test. **(d)** Thymopoiesis (TREC) levels in newborn children in relation to postnatal age. **(e and f)** Intracellular RANKL (in CD4^+^, gdT, and NKT thymocytes; geometric MFI, gMFI) by flow cytometry from thymi of young children (*n* = 13) and shown in relation to estimated numbers of cells per gram of tissue for (e) cTECs or (f) mTECs.

To investigate whether factors affecting thymic output in newborn blood occurred because of the intrinsic effects of thymopoiesis, we assessed thymic production by measuring sjTRECs in *ex vivo* thymic samples obtained from birth up to 2 years of age (Fig. 4 a). To match the analysis made in blood samples, and to decipher the genetic contribution to thymic production, we used a generalized linear model that investigated the contributions made by age (days log_10_ transformed), sex, and *rs2204985* genotype to thymopoiesis. According to the model, postnatal thymic production is significantly influenced by age and *rs2204985* genotype but not by sex (Fig. 4 b). In thymic tissue, the *rs2204985* GG genotype was associated with higher sjTREC levels compared with GA and AA genotypes (Fig. 4 c), suggesting a direct impact of the variant on thymopoiesis that is independent of peripheral T cell homeostasis. As T cell generation rapidly declines with age, we analyzed the correlation between sjTREC levels and age (days log_10_ transformed) and observed a negative correlation already during the first months of life (Fig. 4 d). These findings largely corroborate what is seen in the blood of young children and indicate that blood measurements are reflective of effects within the thymus.

The correlation between RANKL in blood with sjTREC inspired us to look at this protein also within the thymus. We directly analyzed the intracellular expression level of RANKL in CD4^+^, TCRγδ T cells, and NKT by intracellular flow cytometry and related the expression level (geometric mean fluorescence intensity [MFI]) to the numbers of mTECs and cortical TECs (cTECs) per gram of thymic tissue from 13 children previously described (22). This analysis revealed a positive correlation between RANKL and numbers of mTECs, but not cTECs (Fig. 4, e and f). This finding is in line with previously reported roles of RANKL in thymic medulla formation and a possible explanation for its association with thymopoiesis in human newborns.

## Discussion

The concept of a layered hematopoietic development (24) requires a critical assessment of the parameters governing the molecular switches that give rise to the timely evolution of the adaptive immune system in relation to changing needs and environmental exposures. Thymic function is likely to play an important role in this regard, as the need for a large and diverse T cell pool is important for the establishment of healthy immune–microbe interactions early in life (25). For instance, in mice, Tregs produced during the perinatal period have a distinct role that persists during adulthood and maintains self-tolerance (26). Also in mice, intestinal microbes in early life shape the repertoire of PLZF-expressing innate lymphoid cells, impacting disease susceptibility in adulthood (27). In humans, thymic generation of γδ T cells also follow a wave-like pattern during fetal life and infancy (28) with distinct tissue-homing properties (29).

Thymic cross talk between developing thymocytes, TECs, and other stromal cells is critical for constructing the thymic microenvironment during human fetal life (30) and involves lymphotoxin signaling and the RANK-RANKL axis (31). During pregnancy, thymic function transiently involutes, with a decreased cellularity and an increased medullary to cortex ratio. This is a physiological process induced by progesterone, the receptor for which is expressed by TECs (32). Postpartum thymic regeneration in mice is associated with the overexpression of *FOXN1*-regulated genes in TECs (33). Interestingly, in mice, progesterone drives the expansion of natural Tregs through RANK expression in mTECs (34). Here, we provide direct evidence of a positive association between RANKL, LTα, and thymic output in human newborns.

sjTRECs measured in thymic tissue largely reflect thymocytes that have successfully undergone TCRα rearrangement, which occurs at the double-positive (CD4^+^CD8^+^) stage in the thymic cortex, before selection steps. Consequently, thymic sjTREC levels primarily reflect the size and proliferative dynamics of the cortical thymocyte pool and the efficiency of early thymocyte differentiation. In contrast, sjTRECs detected in peripheral blood represent recent thymic emigrants, mature naïve single-positive T cells that have successfully completed both positive and negative selection and exited the thymus. These differences highlight that thymic and blood sjTREC measurements capture distinct stages of T cell development and may therefore be differentially affected by changes in the thymic microenvironment. In this context, the positive association we evidence here between blood RANKL, LTα, and sjTREC levels may reflect an active thymic microenvironment in which thymocyte-derived signals support TEC differentiation and thymic architecture, which in return support thymic production.

Using a large adult cohort, it was identified that in addition to age and biological sex, a common genetic variant (*rs2204985*, A/G alleles) at the *TCRA-TCRD* locus located between *DD2–DD3* gene segments has effects on human thymopoiesis (11), where the G allele was associated with a higher thymic output. Here, we report that this variant is also associated with differences in thymic function in newborns, as assessed by sjTREC levels in both peripheral blood and thymic tissue. Although the precise mechanism by which this genetic polymorphism regulates thymopoiesis remains elusive, there is some evidence for an impact on health and disease. In one study of allogeneic hematopoietic stem cell transplantation (allo-HSCT) recipients from an unrelated donor, the donor *rs2204985* AA genotype was associated with an adverse outcome (35). However, it should be noted that only weak support for an association between *rs2204985* and the outcome of SCT could be found in a cohort of four European populations (36). In an additional study, the *rs2204985* GG genotype was associated with a higher incidence of acute graft-versus-host disease after allo-HSCT, underlining the complexity of immune reconstitution in different transplant setting (37). Finally, in COVID-19 patients with severe pneumonia, the GG genotype at *rs2204985* was associated with improved outcome and a more sustained immune response (38).

Our data also suggest an interplay between the thymic function of mother and child, which could in turn be affected by multiple factors such as maternal health status or inflammatory conditions causing preterm birth, such as preeclampsia, chorioamnionitis, or other microbial infections, and potentially also the *rs2204985* genetic determinant. In this regard, a small fetal thymus as revealed by ultrasound monitoring has been associated with a higher risk of preterm birth (39).

In summary, we identify important factors associated with inter-individual differences in the early life output of T and B cells in human newborns during the first 3 mo of life. Whether these differences translate into functional consequences or altered immune-microbe interactions remains to be determined with longer follow-up and larger cohorts analyzed prospectively.

## Materials and methods

### Inclusion and ethics

The study was performed in accordance with the Declaration of Helsinki, and the study protocol was approved by the regional ethical board in Stockholm, Sweden (DNR: 2014/921-3 and 2016/512-31/1). After obtaining informed consent from parents, blood samples from newborns and parents were collected at the Karolinska University Hospital. Whole blood was frozen directly in EDTA tubes at −80°C. Clinical metadata such as mode of delivery, nutrition, growth, and medications were gathered in a clinical database.

### Mass cytometry

Blood samples obtained longitudinally from children were processed by mixing with an equal amount of stabilizer (Cytodelics AB) (40), incubated for 10 min at ambient temperature and stored at −80°C until further processing. Stabilized samples were thawed, fixed, and lysed using Lysis and Wash buffers (whole blood processing kit; Cytodelics AB). After fixation/lysis of stabilized whole blood samples, 1–2 × 10^6^ cells/sample were plated and cryopreserved using standard cryoprotective solution. For staining, cells were thawed at 37°C, barcoded using an automated liquid handling robotic system (Agilent technologies) using the Cell-ID 20-plex Barcoding kit (Standard BioTools Inc.) as per the manufacturer’s recommendations, and stained batch-wise after pooling. Antibodies targeting the surface antigens are listed in Table 1, washed with cell staining buffer (CSB) (Standard BioTools Inc.), and fixed with 2% formaldehyde, all of which were performed using a custom-built liquid handling robotic platform (41). Cells were then stained with iridium-labeled DNA intercalator at a final concentration of 0.125 mM (MaxPar Intercalator-Ir, Standard BioTools Inc.) on the day of sample acquisition. Following washes with CSB, PBS, and cell acquisition solution (Standard BioTools Inc.), cells were counted and diluted to 500,000 cells/ml containing 0.1× EQ Four Element Calibration Beads (Standard BioTools Inc.) and filtered through a 35-mm nylon mesh. Samples were acquired on a Helios mass cytometer (Standard BioTools Inc.) using CyTOF software version 6.0.626 with noise reduction, a lower convolution threshold of 200, event length limits of 10–150 pushes, a sigma value of 3, and flow rate of 0.045 ml/min.

**Table 1.** Panel of antibodies used for mass cytometry

Tag	Marker	Clone	Vendor
89Y	CD45	HI30	Standard BioTools
113In	CD57	HCD57	BioLegend
115In	HLA-A, B,C	W6/32	BioLegend
141Pr	CD49d	9F10	Standard BioTools
142Nd	CD19	HIB19	Standard BioTools
143Nd	CD5	UCHT2	BioLegend
144Nd	CD16	3G8	BioLegend
145Nd	CD4	RPA-T4	Standard BioTools
146Nd	CD8a	SK1	BioLegend
147Sm	CD11c	Bu15	Standard BioTools
148Nd	CD31	WM59	BioLegend
149Sm	CD25	2A3	Standard BioTools
150Nd	CD64	10.1	BioLegend
151Eu	CD123	6H6	BioLegend
152Sm	TCRgd	5A6.E9	Thermo Fisher Scientific
153Eu	Siglec-8	837535	R&D Systems
154Sm	CD3e	UCHT1	Standard BioTools
155Gd	CD33	WM53	BioLegend
156Gd	CD26	BA5b	BioLegend
157Gd	CD9	SN4 C3-3A2	eBioscience
158Gd	CD34	581	BioLegend
159Tb	CD22	HIB22	BioLegend
160Gd	CD14	M5E2	BioLegend
161Dy	CD161	HP-3G10	BioLegend
162Dy	CD29	TS2/16	BioLegend
163Dy	HLA-DR	L243	BioLegend
164Dy	CD44	BJ18	BioLegend
165Ho	CD127	A019D5	Standard BioTools
166Er	CD24	ML5	BioLegend
167Er	CD27	L128	Standard BioTools
168Er	CD38	HIT2	BioLegend
169Tm	CD45RA	HI100	Standard BioTools
170Er	CD20	2H7	BioLegend
171Yb	CD7	CD7-6B7	BioLegend
172Yb	IgD	IA6-2	BioLegend
173Yb	CD56	NCAM16.2	BD
174Yb	CD99	HCD99	BioLegend
175Lu	CD15	W6D3	BioLegend
176Yb	CD39	A1	BioLegend
191Ir	DNA-Ir	Cell-ID Intercalator-Ir	Standard BioTools
193Ir			
209Bi	CD11b	Mac-1	Standard BioTools

### Antibody staining of cells

The panel of monoclonal antibodies used for this study is indicated in Table 1. Monoclonal antibodies were either purchased pre-conjugated from Standard BioTools or in purified carrier/protein-free buffer formulation from other vendors. Purified antibodies were conjugated to lanthanide metals using the MAXPAR X8 polymer conjugation kit (Standard BioTools Inc.), according to the manufacturer’s protocol. Antibody concentration before and after conjugation was measured by NanoDrop 2000 spectrometer (Thermo Fisher Scientific) at 280 nm. Following conjugation of antibodies, they were diluted 1:1 in Protein Stabilizer PBS (Candor Bioscience GmbH) prior to use in experiments.

### Plasma protein analyses

Plasma samples were gently thawed on ice and centrifuged at 1,500  ×* g*, 4°C for 20 min. 20 μl per sample was transferred to 96-well microtiter plates. Plasma proteins were analyzed using multiplex proximity extension assay technology (Olink Bioscience) as previously described (41). Briefly, each kit consists of a microtiter plate for measuring 92 protein biomarkers in all 88 samples/plate, and each well contained 96 pairs of DNA-labeled antibody probes. Longitudinal samples from each baby were allocated to the same plate to reduce batch-effects related to inter-individual variability. To minimize inter- and intra-run variation, the data were normalized using both an internal control (extension control) and an inter-plate control and then transformed using a predetermined correction factor. The inflammation panel was used for this analysis. The preprocessed data were provided in the arbitrary unit normalized protein expression (NPX) on a log2 scale, where a high NPX represents high protein concentration. Limit of detection (LOD) for each protein was defined as three standard deviations above the background. Protein panels from samples with >10% below LOD values were removed from the analysis.

### Quantification of TREC and B cell receptor excision circle (sjTREC/KREC) and genotyping for the rs2204985 SNP

DNA was extracted from whole blood samples using a Chemagen kit. 500 ng of genomic DNA was preamplified in a 40 μl reaction mix that contained the probes and primers (Eurogentec, Thermo Fisher Scientific) listed in Table S1 and 1× preamplification Master Mix (Standard BioTools) for 10 min at 95°C and then 14 cycles of 95°C for 15 s; 60°C for 4 min with PCR Master Nexus Gradient (Eppendorf). Then sample inlets of 192.24 Dynamic array IFCs (Standard BioTools) were loaded with 3 μl of a mix of 1.8 μl of a 1/20th dilution of preamplified DNA, 2 μl of 2× TaqMan GTXpress (Thermo Fisher Scientific), and 0.2 μl of 2× sample Loading Reagent (Standard BioTools). Assays inlets were loaded with 3 μl of an equal mixture of 2× Assay loading Reagent (Standard BioTools) and 20× Assay that contains the primers and the probe specific for each assay (listed in Table S1). Each assay was loaded in quadruplicate. The IFC was placed into the Juno controller where sample and assay inlets were loaded into the reaction chamber using the Load Mix 192.24 GE program. Real-time PCR data were collected using a Biomark HD instrument after each cycle with the following thermal protocol: 96.5°C for 20 s and 40 cycles of 96°C for 15 s and 60°C for 60 s. All assays were normalized to 150,000 cells using albumin gene quantification and log_10_ transformed.

### Thymocyte isolation

Pediatric thymus samples were obtained from children undergoing cardiac surgery and used according to and with the approval of the French Ministry of Research (Hôpital Necker, Paris, DC-2014-2272). The thymus tissue was mechanically disrupted by cutting the thymic lobes into small pieces and then squeezing the pieces with the plunger of a 5-ml syringe through a 70-μm cell strainer to obtain a single-cell suspension. The cells were then frozen as a dry pellet and stored until further use.

### Quantification of TREC (sjTREC) in thymocytes

DNA extraction was performed using the DNeasy Blood and Tissue Kit (Qiagen). Multiplex real-time quantification was performed using the 7500 Fast PCR system (Life Technologies) in 96-well plates loaded with 20 μl containing 8 μl of DNA (0.5 µg of genomic DNA), 10 μl of 2× Takyon Low Rox Probe MM (Eurogentec), and 2 μl of specific primer-probe mix (Table S2) with the following thermal protocol: 95°C for 10 min and 40 cycles of 95°C for 15 s and 60°C for 60 s sjTRECs were normalized to 150,000 cells using the albumin gene quantification and log_10_ transformed.

### Genotyping for rs2204985 SNP in thymocytes

The *rs2204985* SNP was genotyped in thymic samples using the 7500 Fast PCR system (Life Technologies) in 96-well plates loaded with 10 μl containing 4 μl of DNA (20 ng of genomic DNA), 5 μl of 2× Takyon Low Rox Probe MM (Eurogentec), 0.125 μl of 80× TaqMan Genotyping Assay (Thermo Fisher Scientific), and 0.875 μl of TE Buffer (Thermo Fisher Scientific) according to the manufacturer’s instructions. The following thermal protocol was applied: 95°C for 10 min and 40 cycles of 95°C for 15 s and 60°C for 60 s.

### TEC isolation, flow cytometry analysis, and quantification of TECs

Human TECs were isolated using a Multi Tissue Dissociation Kit and a gentleMACS dissociator according to the manufacturer’s instructions (Miltenyi Biotec). CD45^+^ hematopoietic cells were depleted using anti-human CD45 microbeads (Miltenyi Biotec) to enrich for epithelial cells. Sample preparation and antibody staining were performed using standard procedures. CD45^−^ cells were stained in fluorescence-activated cell sorting (FACS) buffer (PBS containing 0.5% bovine serum albumin and 5 mM EDTA) with the following antibodies: EpCAM BUV737 (EBA-1), DEC205 BV421 (MMRI-7), CD45 PerCP (HI30), and CD31 PE (L133.1) (BD Biosciences). Staining was performed for 30 min at 4°C. Flow cytometry data were acquired using an LSR II Fortessa flow cytometer (BD Biosciences) and analyzed with FlowJo software (BD Life Sciences). TECs were defined as CD45^−^EpCAM^+^CD31^−^ cells. The DEC205 marker was used to segregate cTECs (DEC205^+^) from mTECs (DEC205^−^). Absolute numbers of cTECs and mTECs were calculated from total FACS cell counts and normalized to the weight of thymic tissue (cells per gram) included in the digestion.

### Flow cytometry and quantification of RANKL expression in thymocytes

Sample preparation and antibody staining were performed using standard procedures. Thymocytes were stained in FACS buffer (PBS containing 0.5% bovine serum albumin and 5 mM EDTA) with the following antibodies: TCRαβ BUV737 (IP26), CD4 BUV395 (RPA-T4), CD8 BV785 (SK1), TCRγδ PE-CF594 (B1), Vβ11 BV421 (REA559), CD3 FITC (UCHT1), and Vα24 PE-Cy7 (REA948) (BD Biosciences, Miltenyi Biotec). For intracellular staining of RANKL, cells were fixed, permeabilized, and stained using the FOXP3 Transcription Factor Staining Kit (eBioscience) according to the manufacturer’s instructions. Flow cytometry data were acquired using an LSR II Fortessa flow cytometer (BD Biosciences) and analyzed with FlowJo software (BD Life Sciences). RANKL-expressing cells (TCRγδ^+^, CD4^+^, and NKT) were gated, and RANKL MFI was quantified.

### Online supplemental material

Fig. S1 shows cohort design and TREC analysis description. Fig. S2 shows thymic output in preterm and term delivered children. Fig. S3 shows RANKL and LTα correlation with thymic output and cell population independently of delivery status and steroids administration. Table S1 list of primers and probes used for dosage of sjTREC, cjKREC, sjKREC and genotyping for rs2204985 in newborn’s blood. Table S2 list of primers and probes used for dosage of sjTREC in postnatal thymocytes. Table S3 lists affiliations for the Milieu Intérieur Consortium.

## Supplementary Material

Table S1 List of primers and probes used for dosage of sjTREC, cjKREC, sjKREC and genotyping for rs2204985 in newborn’s blood.

Table S2 List of primers and probes used for dosage of sjTREC in postnatal thymocytes.

Table S3 lists affiliations for the Milieu Intérieur Consortium.

## Data Availability

All code to reproduce analyses and the data necessary to perform the analyses described in the manuscript are available via GitHub: https://github.com/Brodinlab/newborn_TREC/.
